# The complete chloroplast genome of *Fritillaria yuminensis*, a rare and endangered species endemic to China

**DOI:** 10.1080/23802359.2017.1413315

**Published:** 2017-12-07

**Authors:** Yan Li, Zhirong Zhang, Guanghui Lv

**Affiliations:** a Institute of Arid Ecology and Environment, Xinjiang University, Urumqi, China;; b Germplasm Bank of Wild Species in Southwest China, Kunming Institution of Botany, Chinese Academy of Sciences, Kunming, China

**Keywords:** *Fritillaria yuminensis*, chloroplast genome, phylogenetic analysis

## Abstract

ABSTRACT

*Fritillaria yuminensis* is a medicinal species endemic to northwest Xinjiang, China. The complete chloroplast genome sequence of *F. yuminensis* was sequenced by Illumina Hiseq platform. The complete cp genome was 151,813 bp in length, containing a pair of inverted repeats (IRa and IRb) of 26,377 bp, a large single copy (LSC) of 81,533 bp and a small single copy (SSC) of 17,526 bp, respectively. The overall GC contents was 36.9%. A total of 114 genes were annotated, including 80 protein-coding genes, 30 tRNA genes and 4 rRNA genes. A maximum-likelihood phylogenetic analysis indicated that *F. yuminensis* was closely related to *F. tortifolia* and *F. verticillata*.

The perennial herb *Fritillaria yuminensis* (Liliaceae), is a China endemic species distributed in northwest Xinjiang. It was firstly found and published in 1981 (Duan 1981). *F. yuminensis* is a rare medicinal plant. In the recent few decades, it was overly excavated due to low amount and high price, the population size of this rare species reduced dramatically. It has been included in the first class protection plants of Xinjiang Uygur Autonomous Region (Yin et al. 2006). A good knowledge of genomic information of this species would contribute to the study of population genetics and diversity, and the formulation of efficient protection strategy of this endangered resource.

We collected fresh leaves from Tacheng in Xinjiang, China (46°45′N, 82°57′E) and extracted genomic DNA by CTAB method (Doyle 1987). The voucher specimen was deposited at Xinjiang Institute of Ecology and Geography, Chinese Academy of Sciences (XJBI) (accession number WY01623). The DNA material was kept at the Germplasm Bank of Wild Species in Southwest China (GBWSSC), Kunming Institution of Botany, Chinese Academy of Sciences (accession number CBX35-1). A genomic library was built and sequenced by the Illumina Hiseq X-Ten platform (Illumina Inc., San Diego, CA). Sequence filter, contig generation and genome assemble were carried out following Yao et al. (2016) with cp genome of *F. cirrhosa* (GenBank accession number KY646167) as the reference. The complete cp genome was annotated using the Dual Organellar GenoMe Annotator (DOGMA) (Wyman et al. 2004). The annotated cp genome was deposited in GenBank (GenBank accession number MG200070).

About 1.7 Gb raw reads and 164,351 clean reads were obtained, with coverage of 162×. The chloroplast genome was 151,813 bp in length, containing a pair of inverted repeat (IR) regions of 26,377 bp each, a large single-copy (LSC) region and small single-copy (SSC) region with the lengths of 81,533 bp and 17,526 bp, respectively. The overall GC contents of the plastid genome was 36.9%. The cp genome comprised of 114 distinct genes, including 80 protein-coding genes (PCGs), 30 tRNA genes and 4 rRNA genes. Among these genes, 16 genes (*atpF*, *rpoC1*, *rpl2*, *ndhB*, *ndhA*, *petB*, *petD*, *rpl16*, *rps16*, *rps12*, *trnA-UGC*, *trnG-GCC*, *trnI-GAU*, *trnK-UUU*, *trnL-UAA*, *trnV-UAC*) contained one intron, and two genes *(clpP*, *ycf3*) had two introns. Nineteen genes were duplicated, including 8 tRNAs (*trnA-UGC*, *trnI-CAU*, *trnI-GAU*, *trnL-CAA*, *trnN-GUU*, *trnR-ACG*, *trnV-GAC*, *trnH-GUG*), 4 rRNAs, and 7 PCGs (*ndhB*, *rpl2*, *rpl23*, *rps7*, *rps12*, *ycf2*, *ycf15*).

A maximum-likelihood (ML) phylogenetic tree was constructed using the MEGA6.0 (Tamura et al. 2013) with whole cp genome sequence, including 13 *Fritillaria* species and 3 species of *Lilium* as outgroup. The result showed that *Fritillaria* was monophyletic. *F. yuminensis* was closely related to *F. tortifolia* and *F. verticillata* (Figure 1). The complete chloroplast genome sequences of the *F. yuminensis* is useful genomic information to develop variable markers for studying evolution, population genetics and diversity, and the formulation of protection strategy of wild populations of endangered *Fritillaria* species in Xinjiang in future studies.

**Figure 1.**
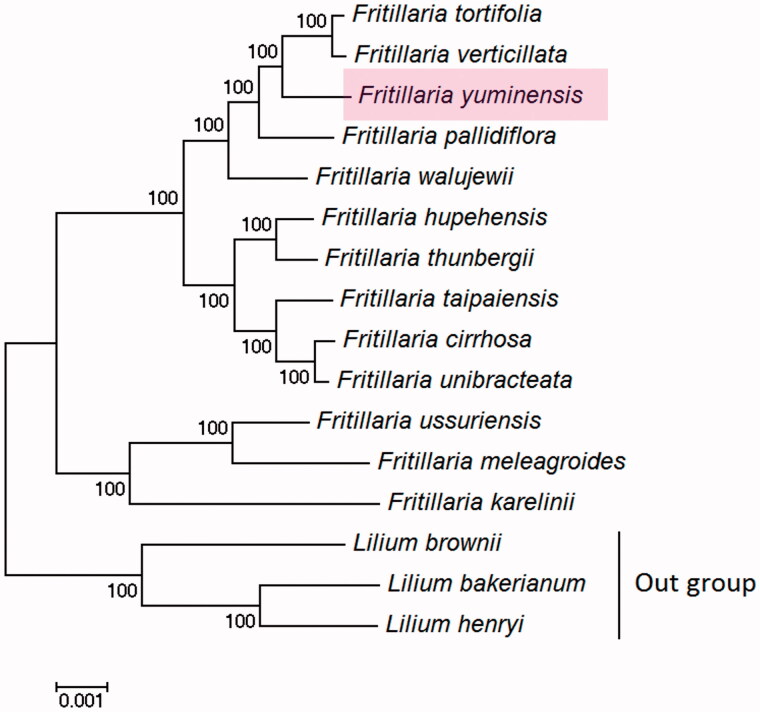
ML phylogenetic tree constructed with complete cp genome sequence. Three *Lilium* species were used as outgroup. The bootstrap values were based on 1000 replicates. GenBank accession numbers: *F. thunbergii* KY646165; *F*. *ussuriensis* KY646166; *F. cirrhosa* KY646167; *F. hupehensis* NC 024736; *F. taipaiensis* NC 023247; *F. unibracteata* KF769142; *F. karelinii* MG211818; *F. tortifolia* MG211819; *F. walujewii* MG211820; *F. meleagroides* MG211821; *F. pallidiflora* MG211822; *F. verticillata* MG211823; *Lilium bakerianum* KY 748301; *L. brownii* KY748296; *L. henryi* NC035570.
